# Hispanic Latin America, Spain and the Spanish-speaking Caribbean: A rich source of reference material for public health, epidemiology and tropical medicine

**DOI:** 10.1186/1742-7622-5-17

**Published:** 2008-09-30

**Authors:** John R Williams, Annick Bórquez, María-Gloria Basáñez

**Affiliations:** 1 Department of Infectious Disease Epidemiology, Faculty of Medicine (St Mary's Campus), Imperial College London, Norfolk Place, London, W2 1PG, UK; 2 Centro Amazónico para Investigación y Control de Enfermedades Tropicales (CAICET) 'Simón Bolívar', Puerto Ayacucho, Estado Amazonas, Venezuela

## Abstract

There is a multiplicity of journals originating in Spain and the Spanish-speaking countries of Latin America and the Caribbean (SSLAC) in the health sciences of relevance to the fields of epidemiology and public health. While the subject matter of epidemiology in Spain shares many features with its neighbours in Western Europe, many aspects of epidemiology in Latin America are particular to that region. There are also distinctive theoretical and philosophical approaches to the study of epidemiology and public health arising from traditions such as the Latin American social medicine movement, of which there may be limited awareness. A number of online bibliographic databases are available which focus primarily on health sciences literature arising in Spain and Latin America, the most prominent being Literatura Latinoamericana en Ciencias de la Salud (LILACS) and LATINDEX. Some such as LILACS also extensively index grey literature. As well as in Spanish, interfaces are provided in English and Portuguese. Abstracts of articles may also be provided in English with an increasing number of journals beginning to publish entire articles written in English. Free full text articles are becoming accessible, one of the most comprehensive sources being the Scientific Electronic Library Online (SciELO). There is thus an extensive range of literature originating in Spain and SSLAC freely identifiable and often accessible online, and with the potential to provide useful inputs to the study of epidemiology and public health provided that any reluctance to explore these resources can be overcome. In this article we provide an introduction to such resources.

## Introduction

The Spanish language is spoken as mother tongue by 300–400 million people, the majority of whom live in the 21 countries around the world where Spanish is the primary language. Yet the public health and epidemiology literature originating in these countries is not readily accessed by peers in the field, who tend more frequently to consult and refer to literature catalogued in English language databases [1,2]. In consequence, many local, national, and regional studies of interest, as well as the possibility of establishing fruitful collaborations in important areas of epidemiology research and public health are missed by the international community [3]. Here we describe and review a number of epidemiological resources from Spain and Spanish-speaking Latin America including the Hispanic Caribbean (SSLAC) -in other islands of the 'Antilles' English, French, and Dutch are spoken. We do not, by any means, aim to provide an exhaustive list, a task that would require far more space than we have at our disposal, but we do attempt to share with the readers a selection that we have found useful in our own professional practice.

The article is organised as follows. We commence by briefly outlining some historical milestones in the development of epidemiology and public health in Spain and of the Universities and Faculties of Medicine in the now predominantly Spanish-speaking countries of Latin America and the Caribbean. Second, we summarise briefly some important early epidemiological research in SSLAC mainly in the area of tropical infectious diseases, and distinctive aspects of the epidemiologies of Spain and SSLAC. Third, and again briefly, we note the results of several bibliometric studies in relation to Spain and SSLAC. Next, we introduce a selection of the Spanish and SSLAC databases available, and a selection of the relevant journals providing tables summarising these (Tables 1 and 2). We conclude by emphasising the range and potential value of the bibliographic resources available and, focusing on SSLAC, discuss the origins of a distinctive approach to the study of public health and the background to an equally distinctive epidemiological context, characteristic features which may merit the attention of workers in the field.

**Table 1:** A selection of health science journals from Spain and Hispano America (part 1)

**Journal [Review system]**	**Abbreviation [ISSN]**	**Area**	**Issues/year**	**Publisher**	**Language**	**Former titles**	**Indexation (where available)**	**Web address**	**Contact details**
*Acta Médica Peruana*	Acta méd peruana	Biomedical science	3	Colegio Médico del Perú, Peru	Spanish & English abstract	-	LATINDEX, LILACS, LIPECS, SciELO		ciromaguina@cmp.or.pe
	[1728–5917 online]								

*Anales de la Facultad de Medicina *[reviewed by editorial board]	An Fac med	Biomedical science	4	Facultad de Medicina San Fernando de la Universidad Nacional Mayor de San Marcos, Peru	Spanish	-	LILACS, LIPECS, SciELO Peru		anales@unmsm.edu.pe
	[1025–5583 print; 1609–9419 online]								

*Anales del Sistema Sanitario de Navarra *[peer reviewed]	An sist sanit Navar	Health and disease; Public health	3	Departamento de Salud. Gobierno de Navarra, Spain	Spanish & English abstract	-	EMBASE, IBECS, IME (Índice Médico Español), LATINDEX, MEDLINE, SciELO		anales@cfnavarra.es
	[1137–6627 print]								

*Anales de Medicina Interna *[reviewed by editorial board]	An Med Interna (Madrid)	Internal medicine	12	Arán Ediciones, S.L., Spain	Spanish & English abstract	-	EMBASE, IBECS, IME, MEDLINE, SciELO		edita@grupoaran.com
	[0212–7199 print]								

*Archivos de Medicina Veterinaria *[peer reviewed]	Arch med vet	Veterinary sciences	3	Facultad de Ciencias Veterinarias, Universidad Austral de Chile, Chile	Spanish & English	-	SciELO, Science Citation Index Expanded, PERIóDICA, and several indexes for veterinary science		archmv@uach.cl
	[0301-732X print; 0717–6201 online]								

*Archivos Españoles de Urología*	Arch Esp Urol	General urology	10	Iniestares, S.A, Spain	Spanish & English	-	EMBASE, IBECS, IME, Index Medicus, MEDLINE, SciELO		urología@archespanoles-de-urologia.es
	[0004–0614 print]								

*Archivos Latinoamericanos de Nutrición (Venezuela)*	[0004–0622]	Medicine; Physiology	4	Sociedad Latinoamericana de Nutrición, Venezuela	Spanish	-	EMBASE, Index Medicus Latinoamericano (IMLA), LILACS, PERIóDICA, SciELO, Science Citation Index Expanded		-

*Boletín chileno de parasitología*	Bol chil parasitol	Parasitology	2	Facultad de Medicina, Universidad de Chile, Chile	Spanish & English abstract	*Boletín de informaciones parasitarias chilenas*; Current title: *Parasitología Latinoamericana*) Official Journal of the Latin American Federation of Parasitologists (FLAP)	Index Medicus, LILACS, MEDLINE, SciELO		See Parasitología latinoamericana
	[0365–9402 print]								

*Boletín de Malariología y Salud Ambiental *[peer reviewed]	BMSA	Tropical medicine; Parasitology; Entomology; Epidemiology; Disease control	2	Instituto de Altos Estudios en Salud Pública "Dr. Arnoldo Gabaldón", Venezuela	Spanish & English abstract	*Boletín de la Dirección de Malariología y Saneamiento Ambiental)*	LATINDEX, LILACS, SciELO		iaespblblio@fundacite.org.gov.ve
	[1690–4648 print and online]								publipeiaesp@yahoo.com

*Ciencia y Enfermería *[reviewed by editorial board]	Cienc enferm	Nursing and Health science	2	Universidad de Concepción, Chile	Spanish & English abstract	-	SciELO		rev-enf@udec.cl
	[0717-2079 print; 0717-9553 online]								

*Colombia Médica *[peer reviewed]	Colomb Med	Health sciences	4	Corporación Editora Médica del Valle, Colombia	Spanish & English	-	BVS, DOAJ, EMBASE, HINARI, ICMJE, IMBIOMED, LATINDEX, MEDLINE, PERIóDICA, REDALYC, SciELO		colombiamedica@gmail.com
	[1657–9534 online]								colombiamedica@yahoo.com

*Enfermedades infecciosas y microbiología clínica *[peer reviewed]	Enferm infecc microbiol clín	Infectious diseases; Medicine	12	Sociedad Española de Enfermedades Infecciosas y Microbiología Clínica, Spain	Spanish & English	*Enfermedades infecciosas*, ISSN 0212–5218; Official Journal of the Spanish Society for Infectious Diseases and Clinical Microbiology	EMBASE, IME, LATINDEX, MEDLINE		doyma@doyma.es
	[0213-005X]								

*Entomotropica *[peer reviewed]	Entomotropica	Entomology in the tropics including Medical entomology	3	Sociedad Venezolana de Entomología, Venezuela	Spanish, English & Portuguese	*Boletin de entomología venezolana *(ISSN 1316–2284)	LATINDEX		editor@entomotropica.org
	[1317–5262 print and online]								

*Gaceta Médica de Caracas *[peer reviewed]	Gac Méd Caracas	General medicine	4	Academia Nacional de Medicina de Venezuela	Spanish & English abstract	-	LILACS, SciELO		ateproca@cantv.net
	[0367–4762 print]			(Editorial Ateproca C.A, Venezuela)					

*Gaceta Médica de México *[peer reviewed]	Gac Méd Méx	General medicine	6	Academia Nacional de Medicina de México, Mexico	Spanish & English abstract	-	ARTEMISA, EMBASE, Index Medicus, LILACS, MEDLINE, PERIóDICA, SciELO		gacetamedica@axtel.net
	[0016–3813 print]								gaceta@medigraphic.com
									gacetamx@starnet.net.mx

*Gaceta Sanitaria *[peer reviewed]	Gac Sanit	Public health; Epidemiology	6	Elsevier Doyma S.L., Spain	Spanish & English	*Gaceta sanitària de Barcelona*	EMBASE, IBECS, Index Medicus, IME, MEDLINE, SciELO		gs@doyma.es
	[0213–9111 print]								

*International Microbiology *[peer reviewed]	Int Microbiol	Microbiology	3	Sociedad Española de Microbiología, Spain	English	*Official Journal of the Spanish Society for Microbiology*	IBECS, LATINDEX, MEDLINE, SciELO		int.microbiol@telefonica.net
	[1139–6709 print]								

*Investigación Clínica *[peer reviewed]	Invest clín	Human and animal biology	3	Instituto de Investigaciones Clínicas, Universidad del Zulia, Venezuela	Spanish & English	-	EMBASE, LILACS, MEDILINE (USA), PERIóDICA		ric_45a@yahoo.es
	[0535–5133 print]								emryder@cantv.net

*Kasmera *[peer reviewed]	Kasmera	Tropical medicine; Microbiology	2	Facultad de Medicina, Universidad del Zulia, Venezuela	Spanish	-	LILACS, SciELO		revistakasmera@hotmail.com
	[0075–5222 print]								bcalvo@telcel.net.ve

*Medicina [Buenos Aires] *[peer reviewed]	Medicina (B Aires)	Clinical and Experimental medicine	6	Fundación Revista Medicina, Argentina	Spanish & English	-	EMBASE, Index Medicus, MEDLINE, SciELO		revmed@intramed.net.ar
	[0025–7680 print]								

*Medifam – revista de medicina familiar y comunitaria*	Medifam	Family medicine; Health care	(ceased publication in 2003)	Sociedad Española de Medicina de Familia y Comunitaria, Spain	Spanish & English abstract	-	EMBASE, IBECS, IME, SciELO		-
	1131–5768 [print]								

*Parasitología Latinoamericana *[reviewed by editorial board]	Parasitol latinoam	Human & animal parasitology	4	Sociedad Chilena de Parasitología, Chile	Spanish & English title	*Parasitología al día *OR *Bol chil parasitol*	LATINDEX, LILACS, PERIóDICA, SciELO		halcaino@uchile.cl
	[0717–7704 print; 0717–7712 online]								

*Research and reviews in parasitology *[peer reviewed]	Res Rev Parasitol	Parasitology	4	Servicio de Parasitología; Centro Nacional de Microbiología, Spain	Spanish & English	Official journal of the Asociación de Parasitólogos Españoles OR *Revista ibérica de parasitología *(ISSN 0034–9623)	EMBASE, LATINDEX		-
	[1133–8466 print]								

*Revista Argentina de Microbiología *[peer reviewed]	Rev Argent Microbiol	Microbiology	4	Asociación Argentina de Microbiología, Argentina	Spanish & English	*Revista de la Asociación Argentina de Microbiología*	EMBASE, LILACS, MEDLINE, PERIóDICA, SciELO)		ram@aam.org.ar
	[0325–7541 print]								

*Revista chilena de enfermedades respiratorias *[reviewed by editorial board]	Rev chil enferm respir	Respiratory diseases	4	Sociedad Chilena de Enfermedades Respiratorias, Chile	Spanish	-	-		revista@serchile.cl
	[0717–5698 print; 0717–7348 online]								

*Revista chilena de infectología *[reviewed by editorial board]	Rev chil infectol	Infection research & reviews; Epidemiology	4	Sociedad Chilena de Infectología, Chile	Spanish & English title	-	LATINDEX, LILACS, MEDLINE, SciELO		revinf@sochinf.cl
	[0716–1018 print; 0717–6341 online]								

**Table 2:** A selection of health science journals from Spain and Hispano America (part 2)

**Journal [Review system]**	**Abbreviation [ISSN]**	**Area**	**Issues/year**	**Publisher**	**Language**	**Former titles**	**Indexation (where available)**	**Web address**	**Contact details**
*Revista chilena de pediatría *[peer reviewed]	Rev chil pediatr	Paediatric research; Public health	6	Sociedad Chilena de Pediatría, Chile	Spanish	-	EMBASE, IMLA, PERIóDICA, SciELO		sochipe@terra.cl
	[0370–4106 print]								

*Revista Costarricense de Ciencias Médicas *[reviewed by editorial board]	Rev costarric cienc méd	Medical science	2	Caja Costarricense de Seguro Social, Costa Rica	Spanish & English abstract	-	DOAJ, LILACS, SciELO		cendeisss@info.ccss.sa.cr
	[0253–2948 print]								

*Revista Costarricense de Salud Pública*	Rev costarric salud pública	Public Health	2	Asociación Costarricense de Salud Pública, Costa Rica	Spanish	-	LILACS, SciELO		dmora@aya.go.cr
	[1409–1429 print]								

*Revista Cubana de Higiene y Epidemiología *[reviewed by Ecimed]	Rev Cubana Hig Epidemiol	Epidemiology; Environmental health	3	Editorial Ciencias Médicas (Ecimed), La Habana, Cuba	Spanish	*Boletín de higiene y epidemiología*	EMBASE, LILACS, SciELO		ecimed@infomed.sd.cu
	[0253–1751 print; 1561–3003 online]								

*Revista Cubana de Investigaciones Biomédicas *[reviewed by Ecimed]	Rev Cubana Invest Bioméd	Biomedical research	4	Editorial Ciencias Médicas (Ecimed), La Habana, Cuba	Spanish	Publicación del Centro Nacional de Información de Ciencias Médicas	BVS Cuba, EMBASE, IMBIOMED, LATINDEX, LILACS, PERIóDICA		ecimed@infomed.sld.cu
	[0864–0300 print]								

*Revista Cubana de Medicina *[reviewed by Ecimed]	Rev Cubana Med	Internal medicine	6	Editorial Ciencias Médicas (Ecimed), La Habana, Cuba	Spanish	Publicación del Centro Nacional de Información de Ciencias Médicas	LILACS, BVS Cuba		ecimed@infomed.sld.cu
	[0034–7523 print, online]								

*Revista Cubana de Medicina General Integral *[reviewed by Ecimed]	Rev Cubana Med Gen Integr	General medicine	6	Editorial Ciencias Médicas (Ecimed), La Habana, Cuba	Spanish & English abstract	*Medicina general integral*	IMLA, LILACS, SciELO		ecimed@infomed.sld.cu
	[0864–2125 print and online]								

*Revista Cubana de Medicina Militar *[reviewed by Ecimed]	Rev Cubana Med Mil	Topics related to military medicine	4	Editorial Ciencias Médicas (Ecimed), La Habana, Cuba	Spanish & English abstract	Publicación del Centro Nacional de Información de Ciencias Médicas	BVS Cuba, LATINDEX		ecimed@infomed.sld.cu
	[0138–6557 print]								

*Revista Cubana de Medicina Tropical *[reviewed by Ecimed]	Rev Cubana Med Trop	Tropical medicine; Microbiology; Parasitology; Epidemiology	3	Editorial Ciencias Médicas (Ecimed), La Habana, Cuba	Spanish & English abstract	Publicación del Centro Nacional de Información de	BVS Cuba, EMBASE, IMLA, Index Medicus, LATINDEX, LILACS,		ecimed@infomed.sld.cu
	[0375–0760 print and online]					Ciencias Médicas	SciELO		

*Revista Cubana de Pediatría *[reviewed by Ecimed]	Rev Cubana Pediatr [0034–7531 print and online]	Paediatrics & related fields	4	Editorial Ciencias Médicas (Ecimed), La Habana, Cuba	Spanish	Publicación del Centro Nacional de Información de Ciencias Médicas	BVS Cuba, EMBASE, Index Medicus, LILACS		ecimed@infomed.sld.cu

*Revista Cubana de Salud Pública *[reviewed by Ecimed]	Rev Cubana Salud Pública [0864–3466 print and online]	Public health & related fields	4	Editorial Ciencias Médicas (Ecimed), La Habana, Cuba	Spanish & English abstract	*Revista Cubana de Administración de Salud*	BVS Cuba, Index Medicus, IMLA		ecimed@infomed.sld.cu

*Revista de Investigación Clínica *[reviewed by editorial board]	Rev invest clín [0034–8376 print]	Clinical medicine & related topics	6	Instituto Nacional de Ciencias Médicas y Nutrición Salvador Zubirán, Mexico	Spanish & English translation published simultaneously	-	ARTEMISA, Index Medicus, LILACS, SciELO		ric@quetzal.innsz.mx

*Revista de la Facultad de Medicina *[reviewed by editorial board]	RFM UCV [0798–0469 print]	Medical science and technology	2	Facultad de Medicina, Universidad Central de Venezuela, Caracas, Venezuela	Spanish & English abstract	-	LILACS, PERIóDICA, SciELO		revista@med.ucv.ve velascom@cantv.net

*Revista de la Facultad de Medicina de la Universidad de Los Andes *[peer reviewed]	MedULA	Health sciences	2	Facultad de Medicina, Universidad de Los Andes, Mérida, Venezuela	Spanish or English with Spanish & English abstracts	-	-		medula@ula.ve

*Revista de la Facultad de Medicina de la Universidad Nacional de Colombia *[reviewed by editorial board]	Rev Fac Med Univ Nac Colomb [0120–0011 print]	Health and health-related fields	4	Universidad Nacional de Colombia, Bogotá, Colombia	Spanish & English abstract	-	IMBIOMED, LILACS, SciELO		revista_fmbog@unal.edu.co

*Revista del Instituto Nacional de Enfermedades Respiratorias *[reviewed by editorial board]	Rev Inst Nal Enf Resp [0187–7585 print]	Respiratory medicine	4	Instituto Nacional de Enfermedades Respiratorias, Mexico	Spanish & English	-	ARTEMISA, EMBASE, IMLA, LILACS, SciELO, PERIóDICA		editoria@iner.gob.mx

*Revista de Salud Pública *[reviewed by editorial board]	Rev salud pública [0124–0064 print]	Public health & related fields	3	Instituto de Salud Pública, Facultad de Medicina, Universidad Nacional de Colombia, Colombia	Spanish & English abstract	-	LATINDEX, LILACS, MEDLINE, SciELO		revistasp_fmbog@unal.edu.com

*Revista Española de Salud Pública *[reviewed by editorial board]	Rev Esp Salud Publica [1135–5727, print]	Public health	6	Ministerio de Sanidad y Consumo, Madrid, Spain	Spanish & English abstract	*Revista de sanidad e higiene pública*	EMBASE, IME, MEDLINE, SciELO		resp@msc.es

*Revista médica de Chile *[peer reviewed]	Rev méd Chile [0034–9887 print; 0717–6163 online]	Clinical medicine; Biomedical science	12	Sociedad Médica de Santiago, Chile	Spanish & English	-	LILACS, MEDLINE, Science Citation Index, SciELO, Social Sciences Citation Index		revmedchile@smschile.cl

*Revista Médica del Instituto Mexicano del Seguro Social *[reviewed by editorial board]	Rev Med Inst Mex Seguro Soc	Medicine	6	Instituto Mexicano del Seguro Social, Mexico	Spanish	-	ARTEMISA		-

*Revista Médica del Uruguay *[peer reviewed]	Rev Méd Urug [0303–3295 print]	Medical science	4	Sindicato Médico del Uruguay, Uruguay	Spanish	-	LILACS, SciELO		rmu@smu.org.uy

*Revista Medica Herediana *[reviewed by editorial board]	Rev Med Hered [1018-130X print; 1729-241X online]	Bio- & social medicine	4	Facultad de Medicina 'Alberto Hurtado', Universidad Peruana Cayetano Heredia, Peru	Spanish, Portuguese & English	-	LILACS, SciELO		rmh@upch.edu.pe

*Revista Panamericana de Salud Pública *OR *Pan American Journal of Public Health *[peer reviewed]	Rev Panam Salud Publica [1020–4989 print]	Public health	6	Pan American Health Organization (PAHO), Washington, USA	Spanish, Portuguese & English	*Boletin de la Oficina Sanitaria Panamericana *OR *Bulletin of the Pan American Health Organization*	EMBASE, LILACS, MEDLINE, SciELO		publiper@paho.org

*Revista Peruana de Medicina Experimental y Salud Pública *[peer reviewed]	Rev Peru Med Exp Salud Publica [1726–4634 print]	Research in experimental medicine; Public health; Nutrition	4	Instituto Nacional de Salud, Peru	Spanish & English abstract	-	LATINDEX, LILACS, LIPECS, SciELO Peru,IMBIOMED,REDALYC		revmedex@ins.gob.pe

*Revista salud pública y nutrición *[peer reviewed]	RESPYN [1870–0160, online]	Epidemiology; Medicine; Public health; Nutrition	4	Universidad Autónoma de Nuevo León, Facultad de Salud Pública y Nutrición, Mexico	Spanish, English & Portuguese	-	ARTEMISA, LATINDEX, LILACS, PERIóDICA, REPIDISCA		respyn@faspyn.uanl.mx pcantu@faspyn.uanl.mx

*Salud Mental *(México)	0185–3325	Medicine; Psychiatry	4	Instituto Mexicano de Psiquiatría "Ramón de La Fuente", Mexico	Spanish	-	ARTEMISA, CLASE, EMBASE, IMBIOMED, LILACS, LATINDEX, PERIóDICA, Social Sciences Citation Index		perezh@imp.edu.mx uribes@imp.edu.mx

*Salud Pública de México *[peer reviewed]	Salud pública Méx [0036–3634 print]	Public health	6	Instituto Nacional de Salud Pública, Mexico	Spanish & English	*Salubridad y Asistencia de México*	ARTEMISA, CLASE, EMBASE, Index Medicus, IME, IMLA, LILACS, MEDLINE, PERIóDICA, SciELO, Social Sciences Citation Index		spm@insp3.insp.mx

*VITAE Academia Biomédica Digital *[reviewed by editorial board]	VITAE [1317-987X online]	Medicine; Biology; Infection; Immunology (mainly reviews)	4	Facultad de Medicina de la Universidad Central de Venezuela, Caracas, Venezuela	Spanish & English abstract	-	LATINDEX		harreche@reacciun.ve

Our aim in presenting this paper is to create an awareness among researchers beyond the boundaries of Spain and SSLAC of the richness and diversity of these resources, and to facilitate their use. We hope that this may: a) help to improve the comprehensiveness and quality of reviews and meta-analyses and minimise possible bias in literature searches; b) widen opportunities for collaborations with workers sharing similar interests, and c) facilitate a fuller understanding of the field in general and of specific areas of interest within it.

## Historical background

### Epidemiology and public health in Spain

Early in the previous millennium the Islamic south of Spain, Al-Andalus, was the centre of scientific and medical knowledge in Europe. In addition, the first universities in Christian Spain (including the present day University of Salamanca), were founded in the 13th C, well before the 15th C completion of the Spanish Reconquest. The beginnings of the study of public health in Spain were seen during the Renaissance under the sponsorship of Philip II [4], in the same era as enormous wealth was flowing into Spain from the Americas. Despite all this, as the 19th C drew to a close, Spain was, in Western European terms, an underdeveloped country characterised by poverty and life expectancy in 1901 of a mere 40 years [5]. Also, despite its colonial history, and unlike Belgium, Germany and the UK, in Spain there had been little development in the area of tropical medicine [6]. However, it was this epoch at the turn of the 20th C that saw the laying down of the foundations of an effective public health system. The Instituto Nacional de Higiene "Alfonso XIII" (INH) had its origin in this period [6], and a National School of Health (Escuela Nacional de Sanidad, ENS) was founded in 1924 [7]. Until the INH and ENS merged in 1934 as the Instituto Nacional de Sanidad (INS), the place of the teaching of 'hygiene' in the Universities had been precarious, being far below the levels seen in other European countries [8]. Unfortunately, the 1936–39 Spanish civil war interrupted this development of the study of epidemiology and public health for some years, and it was not before 1986 that this work began to be revitalised with the reinvention of the INS through the founding of the Instituto de Salud Carlos III [6]. Since then, in recent years (1995–2005) authors based in Spain have accounted for some 2,000 of the papers relating to infectious disease identified in the PubMed database [9]. However a study in 1999 had also identified a further 3,000 papers relating to public health and health policy in Spanish journals indexed in the Spanish Medical Index (Índice Médico Español, IME) [10], and a similar study one year earlier found that 2–3% of the papers indexed in IME related to epidemiology or public health, with only 0.2% published in English [11].

### The University and the teaching of Medicine in Latin America

Pre-dating independence from Spain by nearly three centuries, the early dates of foundation of the first universities in what are now the Spanish-speaking nations of Latin America and the Caribbean, may not be well recognised in the Anglophone world. Claims to be the oldest university in Latin America are controversial, but in 1538 the Universidad Autónoma de Santo Domingo [12] was founded in what is now the Dominican Republic, although not officially recognised until two decades later. In the meantime were founded in 1551 the Universidad Nacional Mayor de San Marcos (UNMSM), Perú [13] and also the Real y Pontificia Universidad de México (RPUM) which, after an interrupted history in the 19th C was to become the Universidad Nacional Autónoma de México (UNAM) [14]. These were followed by the foundation of the Universidad Nacional de Córdoba (Argentina) [15] and the Universidad de Chile [16] in 1622. The oldest medical school in the USA is the University of Pennsylvania School of Medicine, founded in 1765, but the faculties of medicine of UNMSM and UNAM both lay claim to earlier beginnings. Two chairs in medicine were established at UNMSM in 1571, with a functioning faculty by the 17th C, although not formally constituted until 1856. At RPUM the first course on medicine began in 1579 [17] (and, despite the 19th C closures of RPUM, the medical school continued in being until absorbed into the refounded university which became UNAM). In 1676 the Universidad San Carlos de Guatemala also officially opened its doors, incorporating the study of medicine along with theology and law [18].

### Early epidemiological work in Latin America

This long history perhaps reflects the fact that in contrast to the colonisation of Africa and the English Caribbean, primarily focused on trade and the exploitation of resources, in Latin America the plundering of wealth was accompanied by a parallel focus on establishing and peopling a (Catholic) civilisation and conversion of the many indigenous populations [19]. In many aspects this latter focus was a reflection – perhaps even, in a sense, a continuation – of the methods of the Reconquista, i.e. the reconquest of the Iberian peninsula by the Christians after eight centuries of Islamic rule by the Moors, a process that proved a training ground for the conquests in the Americas and which was completed with the fall of Granada in 1492, the very year that Christopher Columbus made his first encounter with the 'New World' [19].

The medical science and epidemiology that grew from this environment in Latin America was faced with the challenge of both autochthonous and imported infections. A surprisingly early record of a correctly made, but overlooked association between biting insects and disease dates back to 1764 and comes from Peru, where the Spanish-born physician Cosme Bueno described both bartonellosis (Carrion's disease) and cutaneous leishmaniasis and attributed it to the bite of small flies called 'uta' (a term still used in the Peruvian highlands to refer to the disease and the sandfly vectors) [20]. The accurate deduction of this relationship preceded the formulation of the 'germ theory' by Pasteur in 1877. From the late 19th C important figures emerge in Latin America, perhaps one of the most prominent being Carlos Finlay in Cuba, who proposed and tried to demonstrate in 1880, while conducting important work on cholera, that mosquitoes transmit yellow fever. In the early 20th C Carlos Chagas in Brazil discovered the presence of *Trypanosoma cruzi *in human blood (the causative agent of what came to be known as Chagas disease) and in 1909 unravelled its transmission by triatomines [21,22]. Rodolfo Robles in Guatemala was the first to hypothesise in 1915 the role of blackflies as vectors of human onchocerciasis (called Enfermedad de Robles or Robles disease in Guatemala) and link the infection to blindness, predating by nearly 10 years the work of Blacklock incriminating *Simulium damnosum *as the vector of river blindness in Africa [23].

Nevertheless, as late as 1960, as Director of the Pan American Health Organization (PAHO), Dr Abraham Horwitz [24] was still able to concur with the observation that in Latin America epidemiology was the "Cinderella of the medical sciences" [25]. Since then epidemiology in Latin America has made substantial advances although in many countries an "epidemiological polarisation" prevails, where communicable diseases persist but chronic diseases occupy a critically important and increasing place [26,27].

### Epidemiology in Spain and Latin America

Spain, since arrival of democracy following the end of the Franco dictatorship in the 1970s, has rapidly achieved a Western European standard of living with research in public health and epidemiology becoming associated with the European tradition [9,28]. In contrast, globally, Brazil and Hispanic Latin America exhibit the highest national levels of health inequality [29-32] reflected in a tradition of research and literature on health inequalities [33] and which is associated with the social medicine movement, a movement having a historical role in attempting to resist both colonialism and post-independence military dictatorships [34,35]. Regrettably there appears to be little knowledge in the English-speaking world of the fruits of this research in the field of social medicine [36]. With regard to this, although Almeida-Filho et al. [33] identified a relative neglect of gender, race, and ethnicity issues in health inequity research in the Latin American literature, they also highlighted, at the methodological level, a remarkable diversity of epidemiological research designs and a refined ecological tradition, with consideration of aggregate and ethnographic methods not evident in other research traditions.

## Bibliometrics and databases

### Bibliometric studies

In public health and epidemiology, Falagas and colleagues [37] reported that between 1995 and 2003, 686 articles (1.4% of the global total) originating from Latin America and the Caribbean were published in Thomson-ISI indexed journals, a number of the same order as for Eastern Europe but far below that of Western Europe or the United States of America. The mean impact factor of 1.7 however was comparable, exceeding that of W Europe (1.5) and approaching that of the USA (2.0). In parasitology, Falagas et al. [38] reported that in the PubMed database over the same period, 17%, of journal articles originated from Latin America and the Caribbean (LAC), comparable with the production from the USA (20%), and that since 2001 increasing production in the former had resulted in LAC displacing the USA from second place behind W Europe (with 35% of the articles). Falagas et al. [39] subsequently reported that LAC assumed third place behind W Europe and Africa in production of articles in the field of tropical medicine with 21% of articles published, ahead of the USA with 11%. However as with parasitology, the mean impact factors were lower -in tropical medicine 0.90 for LAC compared with 1.65 for USA and 1.21 for W Europe. In the fields of microbiology [40] and infectious diseases [41] 1995–2002 productivity in terms of PubMed indexed journals was much lower with just over 2% of articles in both fields although the mean impact factor, 2.89, just exceeded that of W Europe with 2.82, with 3.42 for articles originating in the USA. The latter study also noted that, together with Africa and E Europe, the rate of increase in production in Latin America exceeded that of W Europe and the USA, and that if existing rates of increase were maintained their production would exceed that of the USA within 20 years or so.

**Figure 1:**
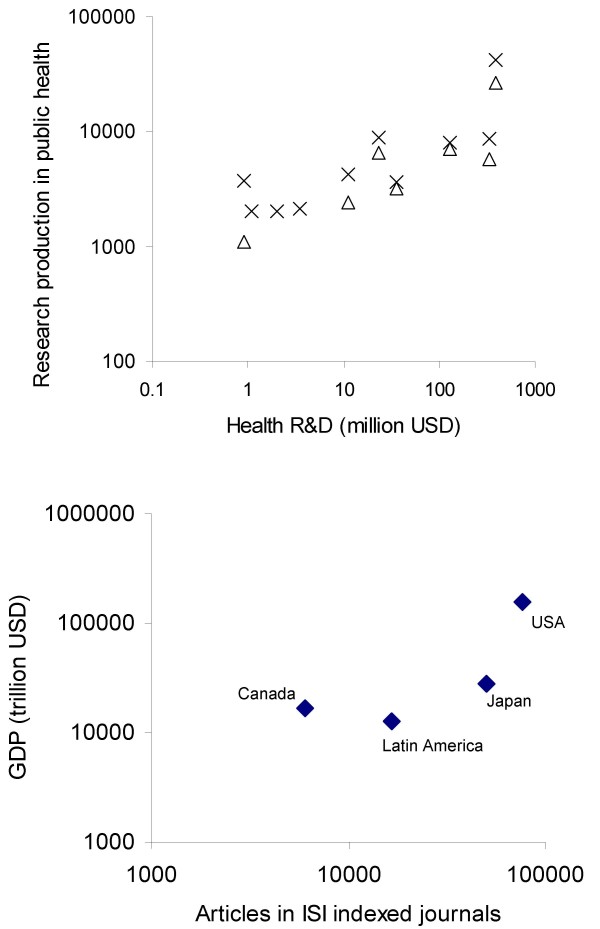
**National expenditure on research and economic performance versus research productivity**. a) Relationship for several Latin American countries between health research expenditure  and: i) journal articles on public health (triangles); ii) total public health research publications (crosses) indexed in LILACS-SP for 1980–2002) [45]. b) Relationship between gross domestic product (GDP) and biomedicine research productivity for three higher income countries and for Latin America (source: Falagas et al [37]).

A recent bibliometric study [33] using both PubMed and the Literatura Latinoamericana en Ciencias de la Salud (LILACS) [42] database noted that observed differences in research production between SSLAC countries could be misleading, e.g. the searching of indexed journals favours Mexico as, for reasons of geography, Mexican researchers engage in relatively more scientific exchange with North American universities. In fact, Hermes Lima et al. [43] point out that in the field of biomedicine, the predominant pattern of collaborations is South-North, favouring North America, rather than strengthening South-South links between countries within Latin America. However it is clear that among dominant factors influencing levels of research publication in public health are, of course, national expenditure on health research and, by implication, the level of economic wealth, an illustration of which is provided in Figure 1.

### Bibliographic databases

The LILACS database is a key resource for the identification of publications originating in Latin America and the Caribbean, whether written in English, Spanish or Portuguese. It includes theses, books and proceedings as well as journal papers. Clark and Castro [1] observed that "LILACS is an under-explored and unique source of articles whose use can improve the quality of systematic reviews" (for a succinct description of LILACS and how to access it see the article by Barreto and Barata in this thematic series [44]). Of 64 systematic reviews published in five high impact factor medical journals, only 2 had used LILACS whereas, of the remaining 62 reviews, 23 restricted their search to English language articles with only 18 clearly specifying no language restriction; for 44 of the reviews a subsequent LILACS search revealed articles which had been omitted but which were suitable for inclusion [1], evidence of the "lost science" highlighted by Gibbs [3]. Between 1980 and 2002, of the 98,000 publications relating to public health indexed in LILACS, Brazil and a group of seven countries of SSLAC (in descending order of production: Chile, Mexico, Argentina, Venezuela, Colombia, Peru and Cuba) each accounted for 42–43% [45]. In these 7 countries of SSLAC the majority (57–89%) of publications were in the form of journal articles with the exception of Peru where 69% was in the form of monographs [45]. Between 94% and > 99% of publications from the SSLAC group, depending on country, were written in Spanish with the majority of the rest in English; Venezuela, with 4%, lead the production of publications in English [45]. Many of the publications in Spanish, however, also had abstracts in English. These publications were to be found in some 400 journals based in Brazil and over 500 journals in SSLAC, although 47% of the articles were located in just 91 journals, of which those publishing the largest number of articles in Spanish were *Revista Médica de Chile *(Chile), *Archivos Latinoamericanos de Nutrición *(Venezuela), *Salud Pública de México *(México), *Gaceta Médica de México *(México), *Revista Chilena de Pediatría *(Chile) and *Revista Médica del IMSS *(México) (see Macias Chapula [45] for the full list of 91 journals and their specialisms).

**Figure 2:**
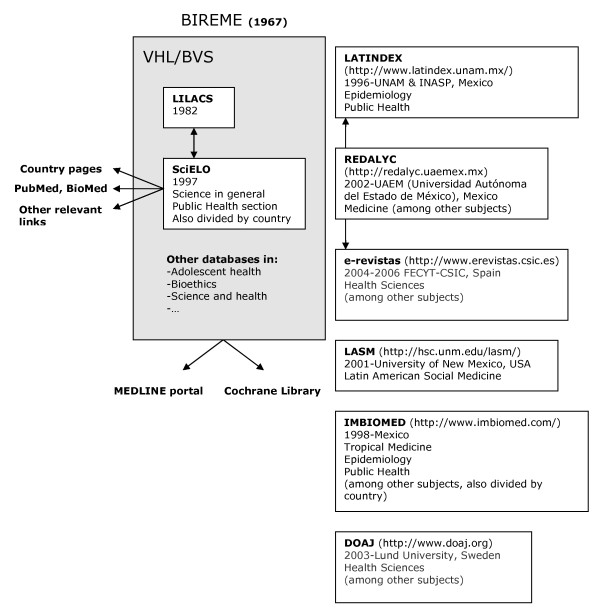
**Databases for Spanish language health journals**. Diagram illustrates databases offering free access to scientific articles with emphasis given to Public Health and Epidemiology journals written in Spanish. Within each box is indicated year of launch, founding institution or organisation, and subjects covered. Arrows represent links between services.

The LILACS database is nested within the Virtual Health Library (VHL) [46] of the Pan-American Health Organization's (PAHO) Latin American and Caribbean Center on Health Sciences Information (BIREME) (Figure 2). The VHL (or BVS, Biblioteca Virtual en Salud [47]/Biblioteca Virtual em Saúde [48]) also encompasses a number of other relevant databases, ADOLEC (Literature on Adolescence Health) and HISA (Latin American and Caribbean History of Public Health) being just two examples. The VHL portal also provides for searches of MEDLINE and the Cochrane database.

Whilst LILACS provides a comprehensive database of Latin American literature, both peer-reviewed and "grey", on public health and epidemiology, the Scientific Electronic Library Online (SciELO) [49], a collaboration between a number or organisations, including BIREME, offers a portal providing free access to many journals from Latin America and the Iberian Peninsula (Spain and Portugal) (see Barreto and Barata [44] in this thematic series for a succinct introduction to this also).

VHL/LILACS is not the only database which specialises in journals emanating from Latin America and/or Spain; see Figure 2. For example, LATINDEX [50] offers a directory of a large number of Spanish and SSLAC journals in all the sciences, a proportion of which appear in a catalogue selected according to international quality standards. There are several others resulting from national or multinational initiatives (e.g. IMBIOMED [51]; LASM (Latin American Social Medicine) [52]) and focusing on medicine and health or with a wider remit (e.g. E-REVISTAS [53]; CLASE [54], PERIÓDICA [54]; REDALYC [55]). Both PERIÓDICA and CLASE were created by UNAM's Centro de Información Científica y Humanística (CICH) in the 1970s and constitute relevant regional sources of information. Regional full text access to articles appearing in the better quality health sciences journals published in Mexico is also available using the CD-ROM based ARTEMISA (Artículos Editados en México de Información en Salud) database or online since 2006 at Medigraphic Literatura Biomédica [56]. Table 3 provides a list of such databases together with the addresses of the relevant websites.

**Table 3:** Bibliographic databases. A selected list of less widely known databases indexing significant numbers of Spanish language articles

Acronym	Spanish	English	Website
ARTEMISA	Artículos Editados en México de Información en Salud	Health Information Articles published in Mexico	[56]

BVS	Biblioteca Virtual en Salud	Virtual Health Library	[46-48]

DOAJ	Directorio de Revistas de Libre Acceso	Directory of Open Access Journals	

HINARI	InterRed-Salud Iniciativa de Acceso a la Investigación	InterNetwork-Health Initiative for Access to Research	

IBECS	Índice Bibliográfico Español en Ciencias de la Salud	Spanish Bibliographic Index in Health Sciences	

IMBIOMED	Índice Mexicano de Revistas Biomédicas Latinoamericanas	Mexican Index of Latin-American Biomedical Journals	[51]

IME	Índice Médico Español	Spanish Medical Index	[10]

IMLA	Index Medicus Latino Americano	Latin American Index Medicus	

LATINDEX	Sistema Regional de Información en Línea para Revistas Científicas de América Latina, el Caribe, España y Portugal	Regional on Line Information System for Latin America, the Caribbean, Spain and Portugal	[50]

LILACS	Literatura Latinoamericana y del Caribe en Ciencias de la Salud	Latin American and Caribbean Health Sciences Literature	[42]

LIPECS	Literatura Peruana en Ciencias de la Salud	Peruvian Health Sciences Literature	

PERIODICA & CLASE	Índice de Revistas Latinoamericanas en Ciencias y Humanidades (de la Hemeroteca Latinoamericana, HELA)	Index of Latin American journals in the sciences & Index of Latin American journals in the humanities	[54]

REDALYC	Red de Revistas Científicas de América Latina y el Caribe, España y Portugal, Ciencias Sociales y Humanidades	Scientific Journals' network from Latin America, the Caribbean, Spain and Portugal, Social Sciences and Humanities	[55]

REPIDISCA	Red Panamericana de Información en Salud Ambiental	Pan American Environmental Health Information Network	

SciELO	Biblioteca Científica Electrónica en Línea	Scientific Electronic Library On Line	

VHL however provides the best starting point for investigating the rich range of Spanish language literature in the field as well as providing a number of other tools such as its "Evidence Portal" and "Health Information Locator". In addition there are many links to national VHL sites which, whilst there is a substantial degree of overlap with the main VHL web site, also provide additional resources – national SciELO sites for example may include journals not featured on the main SciELO site. In fact the wealth of resources is such that it would not be surprising if first-time users experienced a certain degree of bewilderment in navigating their way through this network and, in truth a full description of what is available would stretch to many pages (such a description would also quickly become obsolete as development and consolidation continue to proceed hand in hand).

**Figure 3:**
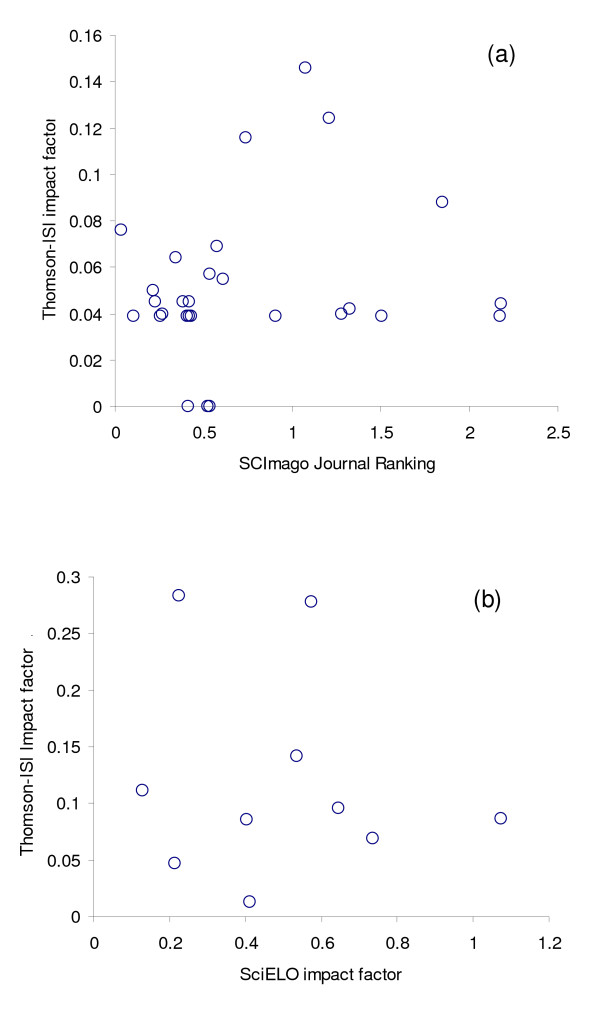
**Scatter plots illustrating lack of consistency between measures of impact**. a) Thomson-ISI impact factors versus SCImago Journal Rankings for 2006 for 28 journals of health and life sciences from Spain and Latin America found in both indexes; b) Thomson-ISI impact factors versus SciELO impact factors for 10 journals of health and life sciences from Spain and Latin America found in both indexes.

Focusing now on the Spanish and Latin American Spanish-language journals themselves, Tables 1 and 2 provide a summary of many of those which we feel may be of use to those working in the field of public health or epidemiology (we should emphasise that this list is by no means exhaustive). Amongst other things Tables 1 and 2 indicate the general area of interest for each journal, frequency of publication and addresses of the web pages, many within the SciELO database, where further details and/or online copies of journal articles may be available (a number of these journals are indexed in widely used databases such as MEDLINE, EMBASE or Ulrich's and links for some are also provided from the websites of the International Committee of Medical Journal Editors [57] or World Association of Medical Editors [58]). While the number of journals specifically focused on public health and epidemiology is not great, many others may potentially be of interest to workers in the field. A significant number of these journals provide an abstract in English and some also in Portuguese (see Tables 1 and 2). Of those focussed on public health, it is worth mentioning a few that have greater visibility (see Table 4). The *Revista de Salud Pública*, published by the Universidad Nacional de Colombia since 1999, treats a wide range of subjects relevant to national as well as international public health. It is indexed in MEDLINE, SciELO, LILACS, LATINDEX as well as in two Colombian databases: the National Index of Scientific and Technological Colombian Journals and LILOCS (Literatura Colombiana de la Salud). In 2006 it had an impact factor on a two year basis of 0.18 and a citation half life of 3.25 years in the SciELO database (Table 4). Two Cuban journals, *Revista Cubana de Higiene y Epidemiología *and *Revista Cubana de Salud Pública *offer slightly different approaches to public health. The first is more empirical and reports findings of studies in environmental hygiene, food-related infections and occupational medicine while the second mainly publishes essay-type articles on historical, controversial or novel issues relevant to public health involving professionals from other fields. The SciELO 2006 impact factor for these journals are 0.1591 and 0.0395 respectively while the half lives are 5.17 and 2.25 years respectively, suggesting that the *Revista Cubana de Higiene y Epidemiología *has more visibility. *Salud Pública de México*, the official journal of the National Institute of Public Health addresses a broad scope of subjects, publishing original articles resulting from research in parasitic diseases' epidemiology to health economics. Its SciELO impact factor for 2006 was 0.2747 and its half life was 4.86 years which reveals its importance in the field. All the articles are available in Spanish and English. Of the Spanish journals, *Gaceta Sanitaria*, *Revista Española de Salud Pública*, and *Anales del Sistema Sanitario de Navarra *provide a robust assortment of information. *Gaceta Sanitaria *is published by the Spanish Society of Public Health and Sanitary Administration (SESPAS) and it has recently been indexed in the Thomson database with an ISI impact factor of 0.825 in 2007 (note the contrast in scale between ISI and SciELO impact factors, reflecting differences in their corresponding bibliometric algorithms; aside from the issue of scale, Figure 3 illustrates disparities between rankings for a selection of journals). *Revista Española de Salud Pública *had an impact factor of 0.0417 and a citation half life of 4.14 years in SciELO in 2006. Although *Anales del Sistema Sanitario de Navarra *is indexed in SciELO, bibliometric information is not provided, however it does have a SCIMago Journal Rank (SJR) of 0.044 (a measure of impact in the SCImago – Science Visualisation – database), while *Gaceta Sanitaria *has an SJR of 0.068 and *Revista Española de Salud Pública *has an SJR of 0.052 in this database. An important source of information of public health in Latin America is the *Revista Panamericana de Salud Pública/Pan American Journal of Public Health *(previously the separate journals: *Boletín de la Oficina Sanitaria Panamericana *and *Bulletin of the Pan American Health Organization*) publishing in Spanish, English, and Portuguese by PAHO since 1997. This publishes original research and analysis, with a focus on health promotion and the evolution of the programmes with which PAHO is involved. Its 2006 SciELO's impact factor was 0.2030 with a citation half life of 4.52 years.

**Table 4:** Public health and epidemiology journals of Spain and Latin America

**Journal**	**Thomson-ISI Impact Factor**	**SCImago Journal Ranking**	**SciELO Impact Factor**
Revista Médica de Chile	0.405	0.2082	0.065
Gaceta Sanitaria	*N/A*	0.068	0.1901
Revista Española de Salud Pública	*N/A*	0.052	0.2105
Revista Panamericana de Salud Pública/Pan American Journal of Public Health	*N/A*	0.092	0.1183
Revista de Salud Pública	*N/A*	0.06	0.0875
Anales del Sistema Sanitario de Navarra	*N/A*	0.044	0.0432
Archivos Latinoamericanos de Nutrición	0.258	0.047	0.0753
Salud Pública de México	*N/A*	0.101	0.2137
Gaceta Médica de México	*N/A*	0.044	*N/A*
Revista Chilena de Pediatría	*N/A*	0.04	0.1250
Revista Médica del Instituto Mexicano del Seguro Social	*N/A*	0.038	*N/A*
Medicina (Buenos Aires)	*N/A*	0.057	0.0556 (2006)
Revista Chilena de Nutrición	*N/A*	0.0	0.2545
Revista Cubana de Higiene y Epidemiología	*N/A*	0	0.1087
Acta Bioquím Clínica Latinoamer	0.125	*N/A*	0.0247
Rev Cubana de Med Gen Integral	*N/A*	*N/A*	0.0392
Revista Cubana de Pediatría	*N/A*	0	0.1125
Revista de Investigación Clínica	0.243	0.055	*N/A*

The table shows those journals having a ranking from at least one of the Thomson ISI, SCImago or SciELO ranking lists

NB **1 **Brazilian journals which also publish articles in Spanish are not included in this table as these are dealt with elsewhere in this issue (see Barreto and Barata [44])

**2 **The Institute of Scientific Information's (ISI) impact factor is calculated using data from the Journal Citation Reports (JCR) from the Science Citation Index (SCI). The algorithm is as follows: the number of times articles published in the former two years were cited in indexed journals during the following year divided by the number of published articles in these two years . The SciELO's impact factor follows the same algorithm but takes citations given by journals indexed in the SciELO database and does it for the former two and three years. The SCImago Journal Rank (SJR) uses a different and more complex algorithm developed by Stanford University for Google named PageRank, the basic concept being that each journal's prestige is dependent on the prestige of the journals citing it which are themselves dependent on the prestige of the journals citing them *et cetera*. It is an iterative process based not only on the number of citations a journal receives but on where these citations come from  and

## Discussion

It is perhaps surprising to witness the quantity and diversity of databases and interfaces devoted to the Spanish language literature in public health and epidemiology, a result of several independent initiatives. In the period from 1996 to 2003 over 10 databases were launched, and although two (LILACS and SciELO) have achieved international recognition, most of this effort appears to have been neglected by the wider international scientific community. Only rarely are these databases used in systematic reviews and citation of articles in Spanish are infrequent in papers in the English language journals of Europe, North America or Australasia.

The multiplicity of resources may be a problem in itself but development is ongoing and some degree of consolidation is likely although the fact that a number of countries are involved, with varying research standards and infrastructure and, indeed, differing policy objectives for public health, will make this task harder and perhaps slow down their adoption by a wider audience. Nevertheless the creation of LILACS and SciELO, robust sources for dissemination of scientific literature, is worthy of remark. Collaborations between Latin American countries have been relatively uncommon, yet these countries now are participating in the expansion of existing initiatives such as VHL/BVS, LILACS and SciELO and links are being developed between independent initiatives such as LATINDEX and REDALYC.

In the context of searching author names in any bibliographic database, it may be worth mentioning in passing a peculiarity of individuals' family names in many Spanish-speaking countries, and one which can impact on the search for publications by a specific author is the use together of paternal and maternal family names (although for everyday purposes only the paternal family name may be given) [59-62]. As an example, let us take the name of an individual from the Spanish-speaking world with perhaps the greatest global 'name recognition', the former Cuban leader Fidel Castro. His full name is Fidel Alejandro Castro Ruz, the paternal family name being 'Castro' and the maternal being 'Ruz'. Were he to be an author of a paper indexed in bibliographic databases, therefore, his name might be cited in different ways in different databases as FAC Ruz, as FA Castro Ruz or as FA Castro, clearly a potential source of confusion.

The increasing interest in disseminating research findings within Spain and Latin America arises in part from the need to keep pace with the global growth of Internet-based resources, but mainly because there is a tradition of research in this field which has been growing in the past few decades [33,63]. The end of the Franco regime in Spain and the emergence of the socialist movements across Latin America can be considered to have provided the sparks for the modern development of public health in these countries, a development which occurred rather late compared to that in other countries. Reintegration into the European mainstream influenced its development in Spain. The influence of specific ideological movements in Latin America meant that it was approached in a somewhat different manner to that in other parts of the world [64]. Much earlier, towards the end of the 18th C the work of Espejo in Ecuador [65-67] and that of Virchow in Europe [68], in the first half of the 19th C, had already provided a basis from which the study of social medicine in Latin America could develop, however the most significant step in the development of this study occurred in Chile in the 1930's with the epidemiological work of the physician and pathologist Dr Salvador Allende [34,69-71]. During the 1960's and 70's the political parties of the left, including that of Allende who was to become President of Chile, integrated health as a priority in their programmes and denounced the role of poverty as a determinant of disease [34,72,73]. The study of social medicine began to expand rapidly and although many experts in the field were forced into exile at the onset of the military dictatorships in the 1970's they continued to contribute from abroad. In those countries with less repressive regimes the development of theory continued to advance the debate [34]. This social medicine approach integrates health and disease in its social, economical and political context and stresses the multi-factorial nature of causality and implies a need for more qualitative research, as well as a variety of study designs and methodologies [33], a distinctive approach to epidemiology which may warrant the interest of the wider international scientific community.

Apart from its distinctive vision of epidemiology, Latin America also represents a very rich and distinctive context which may be of special interest to epidemiologists and other health professionals. Most countries in this region are now experiencing an epidemiological transition characterised by the coexistence of infectious diseases and the so called chronic diseases of "modern life". While, in the second half of the 20th C, huge improvements in public health indicators (e.g. life expectancy and infant mortality) were observed, epidemics of non-communicable diseases began to grow [64]. Globalisation here has been characterised by rapid industrialisation, uncontrolled urbanisation and important changes in lifestyle, all contributing to the emergence of new epidemics but also to the resurgence and/or spread of infectious diseases such as dengue, cholera, and Chagas disease which were considered to belong to the past [64] or be confined to rural areas. Unregulated industrialisation of agriculture characterised by unrestrained release of pesticides and other chemicals not only caused environmental damage but also the appearance of occupational diseases [64]. Two out of the world's ten most populous cities are now found in Latin America, Mexico City and São Paulo, both harbouring over 20 million inhabitants. This accelerated growth was not followed by adequate provision of the basic requirements for human well-being, clean water and sewage disposal. Also, fuelling the burden of non-communicable diseases are changes in nutritional habits and an increasingly sedentary lifestyle. In 2000, 31% of deaths were caused solely by cardiovascular diseases [74], but widespread occurrence of cardiomyopathies arising from Chagas' disease [75] emphasises the overlap between the epidemiologies of chronic and infectious disease. With the highest levels of social inequality in the world [74], Latin America has been facing dramatic increases in violence fostering mental health problems as well as high rates of intentional injuries [74]. Inequities in access to health-care depending on socio-economic status, gender and ethnicity continue to grow [76]. Here we cannot give a comprehensive overview of epidemiology and public health in Latin America, but we wish to remind the reader of its complexity and distinctive nature and of the potentially important contribution that the research undertaken in this region could bring to the international community.

Unfortunately, there is often a perception that Spanish and Latin American journals in the fields of epidemiology and public health are of lower scientific quality. In this same issue, Barreto and Barata [44] comment on the inadequacy of the ISI impact factor to rate foreign language articles on public health and epidemiology and they describe several alternatives proposed by different researchers. The topic is well documented in his article and we will not go over it again, but it is perhaps of interest to note SCImago, employing a recently launched scientometric journal ranking algorithm, developed jointly by researchers from a number of Spanish universities [77]. This project offers an alternative, and perhaps a more appropriate means to judge the soundness of scientific articles, which may be particularly useful in relation to those written from Spanish- and Portuguese-speaking countries. Although the language barrier remains a problem, many journals now provide abstracts in English and, increasingly, journals and databases are encouraging bilingual and multilingual publication.

Dissemination of information about the resources described here is not only important to facilitate global awareness of relevant research and to stimulate the collaboration between Spanish-speaking countries and the international community, but also to encourage and facilitate it within Spain and Latin America, even at the level of individual countries. It has been found that these resources are rarely accessed by researchers in Latin American countries. A study among researchers in 16 countries showed that only 6% of them used LILACS and that after MEDLINE the most accessed interfaces were Google and Yahoo [78]. Institutions in the region rarely provide interfaces such as free access to online libraries and furthermore, in some settings, slow and unreliable Internet connections may take an hour or more to download a single paper, if at all. The price of articles is generally a barrier to the dissemination of scientific literature, which is exacerbated in countries with poor resources and especially in public universities. Open access publishing will certainly make a huge difference, but first requires awareness of the availability of these resources.

In summary, there is much published Spanish language material which is available online, most comprehensively via VHL, LILACS and SciELO. Nevertheless these resources are under utilised, not only by non-Spanish-speaking researchers but also by many researchers based in Spanish-speaking countries. We hope that this article will have contributed to the creation of an awareness of the existence of these resources and that the detailed information provided will facilitate their access.

## Summary

• There is evidence of omission by systematic reviews of relevant studies published in Spanish

• A wealth of bibliographic databases focusing on journals of epidemiology and public health from Spain and Latin America is available

• Historical and present day contexts of public health studies in Spain and Latin America are discussed, emphasising the development of theories of social medicine

• The main features of the most prominent databases are described.

• A detailed list of relevant journals is provided

## Abstracts in non-English languages

The abstract of this paper has been translated into the following languages by the following translators (names in brackets):

• Chinese – simplified characters (Mr. Isaac Chun-Hai Fung) [see Additional file 1]

• Chinese – traditional characters (Mr. Isaac Chun-Hai Fung) [see Additional file 2]

• French (Mr. Philip Harding-Esch) [see Additional file 3]

• Spanish (Dr. María Gloria Basáñez) [see Additional file 4]

## List of abbreviations

ADOLEC: Literature on Adolescence Health database; ARTEMISA: Artículos Científicos Editados en México sobre Salud [Database of science articles on health published in Mexico]; BIREME: Biblioteca Regional de Medicina [Latin American and Caribbean Center on Health Sciences Information]; BVS: Biblioteca Virtual en Salud/Biblioteca Virtual em Saúde [Virtual Health Library]; CICH: Centro de Información Científica y Humanística, UNAM; CLASE: Index of documents published in Latin American journals specialising in the social sciences and humanities; ENS: Escuela Nacional de Sanidad, Spain; E-REVISTAS: Plataforma Open Access de Revistas Científicas Electrónicas Españolas y Latinoamericanas [Open Access Platform for Spanish and Latin American Scientific Electronic Journals]; HISA: Latin American and Caribbean History of Public Health database; IMBIOMED: Índice Mexicano de Revistas Biomédicas Latinoamericanas [Mexican Index of Latin American Biomedical Journals]; IME: Índice Médico Español [Spanish Medical Index]; INH: Instituto Nacional de Higiene "Alfonso XIII", Spain; INS: Instituto Nacional de Sanidad; LAC: Latin America and the Caribbean; LASM: Latin American Social Medicine database; LATINDEX: Sistema Regional de Información en Línea para Revistas Científicas de América Latina, el Caribe, España y Portugal [Regional online information system for Scientific Journals of Latin America, the Caribbean, Spain and Portugal]; LILACS: Literatura Latinoamericana en Ciencias de la Salud [Latin American Literature in Health Sciences]; LILOCS: Literatura Colombiana de la Salud [Colombian Health Literature database]; PAHO: Pan American Health Organization = Organización Panamericana de la Salud (OPS); PERIÓDICA: Índice de Revistas Latinoamericanas en Ciencias [Index of documents published in Latin American journals specialising in science and technology]; REDALYC: Red de Revistas Científicas de América Latina y el Caribe, España y Portugal [Network of Science Journals of Latin America, the Caribbean, Spain and Portugal]; RPUM: Real y Pontificia Universidad de México; SciELO: Scientific Electronic Library Online; SCIMago: Imago Scientae [Science Visualization]; SESPAS: Sociedad Española de Salud Pública y Administración Sanitaria [Spanish Society of Public Health and Health Administration]; SJR: SCIMago Journal Rank; SSLAC: Spanish-speaking countries of Latin America and the Caribbean; UNAM: Universidad Nacional Autónoma de México; UNMSM: Universidad Nacional Mayor de San Marcos, Perú; VHL: Virtual Health Library.

## Competing interests

The authors declare that they have no competing interests.

## Authors' contributions

JRW conceived the paper; AB and M-GB helped prepare figures and tables; AB drafted the discussion; all authors contributed to the researching of the databases and the writing of the paper, and read and approved the final version submitted.

## Supplementary Material

Additional file 1 Chinese abstract – simplified characters. Translation of the English abstract into Chinese using simplified characters. Click here for file

Additional file 2 Chinese abstract – traditional characters. Translation of the English abstract into Chinese using traditional characters. Click here for file

Additional file 3 French abstract. Translation of the English abstract into French. Click here for file

Additional file 4 Spanish abstract. Translation of the English abstract into Spanish. Click here for file
