# Supplementary material for: Hispanic Latin America, Spain and the Spanish-speaking Caribbean: A rich source of reference material for public health, epidemiology and tropical medicine
Source: Emerg Themes Epidemiol. 2008 Sep 30;5:17. doi: 10.1186/1742-7622-5-17 (PMC2584035; doi:10.1186/1742-7622-5-17)
Supplement: Additional file 1 — Chinese abstract – simplified characters. Translation of the English abstract into Chinese using simplified characters. [file 1742-7622-5-17-S1.pdf]

Simplified Chinese / 简体中文

分析透视

西班牙语拉丁美洲、西班牙及西班牙语加勒比海地区：一个丰富的公共卫生、流行病学及热带病学参考文献来源

作者：John R Williams, Annick Bórquez, Maria Gloria Basanez

摘要

西班牙及使用西班牙语的拉丁美洲与加勒比海地区出版诸多与流行病学及公共卫生有关的健康科学期刊。西班牙的流行病学科研主题与其邻国有许多共同特点，而拉丁美洲的流行病学在众多方面富有当地特色。另外，在流行病学及公共卫生研究方面，其独特的理论与哲学方法则源于一些尚未受到足够注意的诸如拉丁美洲社会医学运动的传统。在一些专门收录西班牙及拉丁美洲健康科学文献的在线文献目录数据库中，其中尤以拉丁美洲卫生科学文献 (Literatura Latinoamericana en Ciencias de la Salud, LILACS) 及 LATINDEX 最为著名。有些如 LILACS 者则广泛收录灰色文献。文献库除使用西班牙语界面外，亦有提供英语及葡萄牙语界面。文章亦有提供英文摘要的，而愈来愈多期刊开始出版英文文章。提供全文免费下载的文章变得易于访问，其中最齐全的来源是科学电子图书馆在线 (Scientific Electronic Library Online, SciELO)。因此，只要克服了不愿意运用这

些资源的心态，读者就可免费检索和网上访问这一广泛系列的源于西班牙及使用西班牙语的拉丁美洲与加勒比海地区的文献资源，充分利用并整合其流行病学及公共卫生研究的有用信息。这篇文章仅对这些资源作一引介。

（中文摘要翻译：冯俊熙）
