# Supplementary material for: Hispanic Latin America, Spain and the Spanish-speaking Caribbean: A rich source of reference material for public health, epidemiology and tropical medicine
Source: Emerg Themes Epidemiol. 2008 Sep 30;5:17. doi: 10.1186/1742-7622-5-17 (PMC2584035; doi:10.1186/1742-7622-5-17)
Supplement: Additional file 2 — Chinese abstract – traditional characters. Translation of the English abstract into Chinese using traditional characters. [file 1742-7622-5-17-S2.pdf]

Traditional Chinese / 繁體中文

分析透視

西班牙語拉丁美洲、西班牙及西班牙語加勒比海地區：一個豐富的公共衛生、流行病學及熱帶病學參考文獻來源

作者：John R Williams, Annick Bórquez, Maria Gloria Basanez

摘要

西班牙及使用西班牙語的拉丁美洲與加勒比海地區出版諸多與流行病學及公共衛生有關的健康科學期刊。西班牙的流行病學科研主題與其鄰國有許多共同特點，而拉丁美洲的流行病學在衆多方面富有當地特色。另外，在流行病學及公共衛生研究方面，其獨特的理論與哲學方法則源於一些尚未受到足夠注意的諸如拉丁美洲社會醫學運動的傳統。在一些專門收錄西班牙及拉丁美洲健康科學文獻的在線文獻目錄數據庫中，其中尤以拉丁美洲衛生科學文獻 (Literatura Latinoamericana en Ciencias de la Salud, LILACS) 及 LATINDEX 最爲著名。有些如 LILACS 者則廣泛收錄灰色文獻。文獻庫除使用西班牙語界面外，亦有提供英語及葡萄牙語界面。文章亦有提供英文摘要的，而愈來愈多期刊開始出版英文文章。提供全文免費下載的文章變得易於訪問，其中最齊全的來源是科學電子圖書館在線 (Scientific Electronic Library Online, SciELO)。因此，只要克服了不願意運用這

些資源的心態，讀者就可免費檢索和網上訪問這一廣泛系列的源於西班牙及使用西班牙語的拉丁美洲與加勒比海地區的文獻資源，充分利用並整合其流行病學及公共衛生研究的有用信息。這篇文章僅對這些資源作一引介。

（中文摘要翻譯：馮儁熙）
