# Supplementary material for: Hispanic Latin America, Spain and the Spanish-speaking Caribbean: A rich source of reference material for public health, epidemiology and tropical medicine
Source: Emerg Themes Epidemiol. 2008 Sep 30;5:17. doi: 10.1186/1742-7622-5-17 (PMC2584035; doi:10.1186/1742-7622-5-17)
Supplement: Additional file 3 — French abstract. Translation of the English abstract into French. [file 1742-7622-5-17-S3.pdf]

French / Français

Perspectives analytiques

L’Amérique latine hispanophone, l’Espagne et les Caraïbes hispanophones : une grande ressource de documents de référence dans les domaines de la santé publique, de l’épidémiologie et de la médecine tropicale.

Auteurs : John R Williams, Annick Bórquez et María Gloria Basañez.

Résumé

Il existe une multiplicité de revues en sciences de la santé provenant d’ Espagne et des pays hispanophones d’ Amérique latine et des Caraïbes qui sont d’intérêt en épidémiologie et en santé publique. Tandis que le sujet de l’épidémiologie en Espagne partage en grande part les mêmes caractéristiques avec ses voisins en Europe occidentale, en Amérique latine plusieurs aspects de l’ épidémiologie sont particuliers à cette région. Il existe également différentes approches dans la théorie et la philosophie de l’étude de l’épidémiologie et de la santé publique, nées de traditions telles que le mouvement de médecine sociale en Amérique latine, qui ne sont pas bien connues à l’échelle mondiale. Plusieurs bases de données bibliographiques sont disponibles en ligne qui se concentrent principalement sur la littérature en santé publique provenant d’Espagne et d’ Amérique latine, dont les plus importantes sont

Literatura Latinoamericana en Ciencias de la Salud (LILACS) et LATINDEX. Certaines bases de données, comme LILACS, indexent une quantité considérable de littérature grise. Les interfaces sont disponibles non seulement en espagnol mais en anglais et en portugais. Les résumés d'articles sont parfois disponibles en anglais. Un nombre grandissant de revues ont commencé à publier des articles entièrement écrits en anglais. Des articles disponibles gratuitement en texte intégral commencent à être disponibles, une des sources les plus complètes étant la *Scientific Electronic Library Online* (SciELO). Un grand choix de littérature provenant d'Espagne et des pays hispanophones de l'Amérique latine et des Caraïbes est donc librement identifiable et souvent disponible en ligne, pouvant potentiellement apporter des contributions utiles à l'étude de l'épidémiologie et de la santé publique, à condition de surmonter toute réticence à l'exploration de ces ressources. Cet article propose une introduction à ces ressources.

Traduit de l'anglais par Philip Harding-Esch
