# Supplementary material for: Hispanic Latin America, Spain and the Spanish-speaking Caribbean: A rich source of reference material for public health, epidemiology and tropical medicine
Source: Emerg Themes Epidemiol. 2008 Sep 30;5:17. doi: 10.1186/1742-7622-5-17 (PMC2584035; doi:10.1186/1742-7622-5-17)
Supplement: Additional file 4 — Spanish abstract. Translation of the English abstract into Spanish. [file 1742-7622-5-17-S4.pdf]

Spanish / Español

Perspectivas analíticas

España y los países hispanohablantes de Latinoamérica y el Caribe: una fuente fértil de recursos de referencia en salud pública, epidemiología y medicina tropical.

Autores: John R Williams, Annick Bórquez and Maria Gloria Basanez

Resumen

En el área de ciencias de la salud relevantes a la epidemiología y salud pública, existe una multiplicidad de publicaciones periódicas que se originan en España y en los países hispanohablantes de Latinoamérica y el Caribe de las cuales se tiene un conocimiento escaso fuera de la región. Mientras la disciplina de la epidemiología en España comparte muchas similitudes en cuanto a su práctica y características con el resto de Europa occidental, ciertos otros aspectos son propios y únicos de Latinoamérica, ameritando un tratamiento por separado. En particular, dicha región ha aportado enfoques teóricos y filosóficos distintivos como resultado del movimiento de medicina social, del cual existe un conocimiento limitado fuera del ámbito latinoamericano. Varias bases de datos con énfasis en la literatura de ciencias de la salud que se genera en España y en Latinoamérica se encuentran actualmente disponibles en línea en la red, hallándose entre las más prominentes las de la Literatura Latinoamericana en Ciencias de la Salud (LILACS) y LATINDEX. La primera en particular contiene un gran número de referencias de difícil acceso tales como informes, monografías, y otros

documentos de la denominada ‘literature gris’. Aparte del español, es común encontrar que las bases de datos contienen portales e interfases en ingles y en portugués, con un número creciente de resúmenes y artículos publicados en inglés. Asimismo, se está haciendo cada vez más frecuente que artículos enteros puedan bajarse de la red en forma gratuita, destacándose la Biblioteca Científica Electrónica en Línea (SciELO) como una de las fuentes principales. Concluimos que la literatura especializada en epidemiología y salud pública proveniente de España y los países hispanohablantes de Latinoamérica puede aportar en gran medida al estudio y práctica de estas disciplinas a un nivel más amplio siempre y cuando pueda superarse cualquier renuencia a explorar la utilidad de dichos recursos, los cuales nos proponemos a introducir en el presente artículo.

Traducido por María-Gloria Basáñez
